# Experiences of Tobacco Smoking and Quitting Among Mental Health Consumers

**DOI:** 10.1111/hex.70377

**Published:** 2025-08-10

**Authors:** Helena Roennfeldt, Marianne Wyder, Coral Gartner, Alice Holland, Norah Elvidge, Dan Siskind, Cheneal Puljević

**Affiliations:** ^1^ Metro South Health Addiction and Mental Health Services Brisbane Australia; ^2^ School of Health Sciences and Social Work Griffith University Brisbane Australia; ^3^ NHMRC Centre of Research Excellence on Achieving the Tobacco Endgame, School of Public Health The University of Queensland Brisbane Australia; ^4^ Faculty of Medicine The University of Queensland Brisbane Australia

**Keywords:** consumer experience, mental health, recovery, smoking cessation

## Abstract

**Background:**

Rates of smoking remain high in people who have a diagnosis of mental illness. The high prevalence of smoking in this population highlights the need to engage people experiencing mental ill‐health in enhancing quit‐smoking programs.

**Method:**

This study examined the experience of tobacco smoking, reasons for and benefits of quitting smoking among people diagnosed with a mental illness. We conducted in‐depth, semi‐structured interviews with 17 participants who had attempted to quit smoking to gain insights into their experiences and gather recommendations for improved smoking cessation support.

**Results:**

Findings indicate a link between smoking and mental health, with consumers using smoking as a way to cope with psychological distress. Often, the reasons for quitting smoking were associated with increased personal recovery.

**Conclusion:**

This study highlights the role of support and the right timing to maximise consumers' likelihood of quitting smoking. Smoking cessation interventions should be delivered in a recovery‐focused way, which enhances self‐determination and the personal decision to quit smoking.

**Patient or Consumer Contribution:**

The first author is in a designated lived experience (Consumer) role. The first author conducted the interviews and was explicit regarding their lived experience of mental health challenges and experience as an ex‐smoker when engaging with participants.

## 1 Introduction

Globally, tobacco smoking is a leading cause of mortality and morbidity and contributes to significant health inequalities [1, 2]. While smoking has declined in the general Australian population over recent decades, it remains prevalent in people who have a diagnosis of mental illness [3, 4, 5, 6]. Higher prevalence of smoking seems related to attempts to relieve higher psychological distress and symptoms of mental illness, which may be related to false beliefs that smoking helps reduce stress [7].

Studies show that compared with the general population, those with a diagnosis of mental illness experience higher levels of nicotine dependence, higher levels of smoking‐related illness, and lower rates of quitting [7]. Reducing smoking among people with a diagnosis of mental illness, therefore, is a high priority to increase average life expectancy for this population, which is currently 15 years lower than that of the general population [8]. While many factors contribute to higher premature mortality and morbidity among this population, smoking is considered to be a major contributor [6]. Furthermore, people with a diagnosis of mental illness, who are already a financially disadvantaged population, experience added financial burden from smoking [9, 10], especially in countries such as Australia, which has a high tobacco excise tax rate [11].

People who have a mental health diagnosis are as motivated to quit as the general population [12]. However, despite efforts to integrate smoking cessation support into routine mental health treatment, for a variety of reasons, smoking cessation support is less likely to be offered and remains underutilised [13, 14, 15]. While people who experience mental health concerns have higher levels of nicotine dependence and may experience more severe withdrawal symptoms, they are also less likely to be able to afford smoking cessation medicines, such as nicotine replacement products [16, 17]. Furthermore, this population group also face additional barriers to quitting, such as limited social support, social norms that support smoking, high financial stress, and social and economic disadvantage [18].

The high prevalence of smoking in this population highlights the urgent need for targeted smoking cessation interventions and concerted efforts to engage people experiencing mental ill‐health in quit‐smoking programs [6, 19]. This study explored the facilitators and barriers to quitting smoking among people with lived experiences of mental illness and smoking, to assist with developing targeted interventions.

## 2 Methods

### 2.1 Study Design

This qualitative study employed in‐depth, semi‐structured individual interviews to understand participants' experiences of smoking and their attempts and successes in quitting smoking. The aim was to develop recommendations for optimised smoking cessation support. Queensland Health provided ethics approval [HREC/2020/QMS/70453].

### 2.2 Participants

People over the age of 18 who had a diagnosis of a mental illness and had made an attempt to quit smoking in the last 5 years were eligible to take part in the study. Participants were recruited from the outpatient services of Metro South Health Services and a community mental health service in Queensland, Australia, through self‐referral in response to recruitment flyers, discussions with treating clinicians, or attending an information session at the community mental health service, where the lead author spoke with consumers attending this service about the study. Potential participants contacted the lead author directly to indicate interest in participation and were screened for eligibility. Participants received a $30 gift card in recognition of their expertise and time.

### 2.3 Data Collection

A semi‐structured interview guide was developed by the authors based on existing literature [20, 21]. All participants were asked to describe their experience with smoking, any benefits they experienced when they quit smoking, their experiences of previous quit attempts, and, when appropriate, why they had recommenced smoking. Participants were also asked to share their recommendations for improved smoking cessation support. Data collection occurred in 2023. Interviews were conducted in person and audio‐recorded with participant consent. The interviews were transcribed verbatim and then deidentified by the lead author. Participants were given a pseudonym to maintain their anonymity.

### 2.4 Data Analysis

Data analysis was guided by reflexive thematic analysis (RTA), outlined by Braun and Clark [22]. An inductive approach was used, with analysis guided by the data rather than a preconceived framework [23] or hypothesised themes. The first and second authors read the transcripts several times to familiarise themselves with the data. The initial codes were generated by HR and reviewed by MW. Potential patterns and overarching themes were then formulated based on the meaning present across the data [23]. The data was analysed using NVIVO 1.6 software (2022).

## 3 Results

### 3.1 Participant Characteristics

The final sample consisted of 17 participants, comprising nine males and eight females. Following the inclusion criterion, all participants had quit smoking or attempted to quit smoking in the past 5 years. Participants reported that they began smoking between the ages of eight and 25, with a mean age of 16.5 years. Eight of the participants had resumed smoking cigarettes.

## 4 Relationship Between Mental Health and the Experience of Smoking

In sharing their experiences of smoking, all participants described a strong relationship between smoking and managing their experiences of psychological distress. Therefore, a central theme throughout the findings was the relationship between mental health and the experience of smoking, including the reasons for starting smoking, perceived benefits of smoking, and reasons for quitting. Under this overarching theme, several sub‐themes were identified, including: smoking to cope with negative experiences and emotions, experiences of connection and segregation, and the complex mix of negative and positive impacts of smoking. Recommendations are also provided in the theme of integrated support within holistic and personal growth.

### 4.1 Smoking to Cope With Negative Experiences and Emotions

For many, starting smoking was described as strongly intertwined with a way of coping with negative emotions and experiences related to a mental health diagnosis or entering the mental health system. All participants perceived specific mental health benefits of smoking, most notably managing feelings of anxiety and stress. Smoking was considered dependable in alleviating distress, and for some participants, it became a trusted way of coping.

It also helped a lot with the anxiety that I was feeling. I was very nervous and struggling after being in the hospital for quite a lengthy period of time, and I needed something to help with those emotions that I found really overwhelming.

(Cameron)

It helps me deal with my emotions, and they are not going to hurt me. You know, I know it's bad for my lungs and everything, but it's not hurting me in that moment.

(Rowan)

Consequently, giving up smoking was considered harder because smoking was associated with mental health benefits.

Because I have always suffered from anxiety and depression, and my anxiety would get so much worse after a couple of days of not smoking. I'm not sure, I suppose my body is so used to having a cigarette, but I am really susceptible to anxiety, and I think that makes it harder for a lot of smokers with mental illness.

(Taylor)

Some described continuing to smoke while being aware of the physical harms caused by smoking as reflecting their feelings of shame and low self‐worth.

And let's face it, to do that sort of abuse to yourself, you've got to be in a state where you no longer have positive regard for yourself.

(Drew)

A part of me didn't care about the damage I might be doing to my body. Because of the shame stuff, because I'm not worth having a healthy body or a long life anyway.

(Ash)

The experience of smoking was described by participants as easing anxiety and reflecting feelings of low self‐worth. Smoking was seen as a way to manage mental health struggles, which added to the challenges of quitting. Therefore, smoking was a physical addiction with a substantial psychological component.

### 4.2 Experiences of Connection and Segregation

Most participants also described smoking as a way of being able to connect with others, as the act of smoking created opportunities for social interaction. Smoking was also seen as a way to establish a routine and social connection, which was missed when people tried to quit.

It is funny when you're outside a hotel having a smoke or whatever, and some stranger starts talking. Smokers become more sociable because you and I don't know each other, but we wouldn't be having this conversation right now if it wasn't to do with smoking.

(Morgan)

I didn't have a reason to get out of bed, and got tired of lying around all day, and started smoking again. Especially first thing in the morning, it was always the hardest.

(Riley)

Alternatively, other participants spoke about how government and social restrictions associated with smoking made them feel separated from others, or that members of the public perceived them negatively because of their tobacco smoking. These participants remarked that these social prohibitions contributed to lowering the enjoyment they attached to smoking. While these participants described how these social restrictions negatively impacted their lives while smoking, none of them described this as the main driving force behind quitting. However, they did describe the positive impacts and freedom associated with quitting.

I could watch a movie without leaving and fly on a plane. I couldn't do these things before because I had to leave to smoke. I couldn't go out to dinner with people. It was just too hard

(Cameron)

Notably, the social stigma and personal shame they felt associated with smoking paralleled and sometimes compounded the stigma experienced related to their mental health.

You're almost like an outsider these days. Back in the day, you used to have a group of smokers, whereas now you are usually by yourself because there's hardly anywhere you can smoke. We got to go all the way out there, or yeah, so basically, smokers are like second‐class citizens or outcasts. It used to be the ‘in thing’. Yeah, now it is like I'm an outcast.

(Taylor)

The growing segregation got harder and harder. Like, you know, really noticing that you had to walk further and further before you could find somewhere to smoke and that people would start throwing you greasy looks and making rude noises when you smoke.

(Ash)

The association between smoking and social connection reflected an opportunity to bond with others who smoke. However, segregation associated with smoking compounds the existing stigma associated with having a mental health diagnosis.

### 4.3 A Complex mix of Positive and Negative Experiences Related to Tobacco Smoking

Although smoking was associated with perceived positive emotional or mental health benefits, participants also described negative impacts of smoking, most notably on their health, finances and social lives. For many, the fear associated with the negative physical consequences of smoking was felt acutely and was attributed as a driver to quit smoking.

I had pneumonia, and I had X‐rays done…the doctor says all these signs of emphysema are starting. So that was another scare…you'd see, like, the amputees like they've had, they've had a leg cut off because of gangrene or something from smoking. Yeah. And then down at the smoking area, having a cigarette. Or I'd see people in the hospital. Yeah, I see people in the hospital on trolleys with tubes and stuff coming out of them. And I'm thinking, “Wow, I don't want to get to that.”

(Finlay)

For many, the experience of smoking was often described as a complex mix of pleasure, relief and negative physical sensations.

I didn't like the effect it had on my body, the fact that I struggled with breathing and would hyperventilate. I also felt quite fatigued, and I didn't enjoy that. I really hated the smell of smoking. It's probably one of the biggest things that I disliked about it. And then it was such an obvious smell to both me and to other people. I remember the smell of my fingers when I was going to bed at night. I remember the smell of my clothes and hair, which I found unpleasant. But I really liked that it helped me manage how anxious I was.

(Cameron)

The financial impact of smoking meant that many participants stated that they had prioritised smoking over other essential items such as food, bills and rent. However, none mentioned that this was the main incentive for them to quit smoking.

It was the first thing. I would get less food. I would not pay my bills. I'd be late with my rent. Cigarettes came first.

(Ash)

The financial cost of purchasing tobacco could also put some participants at risk of further adverse health and social effects by resorting to smoking other people's discarded butts. One person also commented that he found himself prioritising smoking over his friendships.

Picking up the durries on the side of the road is nothing, you know, like if you ask anyone who's been homeless, it's like diving into a rubbish bin to get a meal, that's nothing.

(Tracey)

I was using all my friends for cigarettes, and you know I'd go to a person's house just in the hope that they have a cigarette there so I can get my cigarette. I wouldn't see a person, I would see a potential cigarette.

(Ainsley)

I've seen people offer sex for cigarettes, and I have had to smoke people's butts because I didn't have any cigarettes or money

(Ash)

For participants, smoking had sometimes contradictory impacts. Negative impacts contributed to the participants' motivation to quit; however, negative impacts alone were often not perceived as sufficient reasons to quit.

### 4.4 Benefits of Quitting

Significantly, all participants, regardless of whether they had quit for good or had recommenced smoking, agreed that life, is or was, better without cigarettes. Despite the benefits gained through smoking, on balance, they felt like life was better when not smoking. Participants experienced benefits related to improved finances, physical health, mental health and social connections.

## 5 Desired Cessation Support and Recommendations

Participants reflected that to be able to quit smoking, it needed to be the right time when their level of distress had eased, and it was necessary to have the right support and strategies in place. When participants had received smoking cessation support, these were described as only targeting their physical dependency on nicotine, and the cost of nicotine replacement therapies was prohibitive and, as such, inaccessible. Some participants mentioned the potential benefits of combination NRT and expressed support for its use. Currently, combination and extended NRT are not included in the Pharmaceutical Benefits Scheme (PBS) in Australia. However, there is evidence indicating their effectiveness in populations with high dependency [13].

### 5.1 Integrated and Holistic Support for Personal Growth

Desired support included psychological interventions, and participants spoke about how they had received little support from health care workers in understanding the role smoking played in managing their mental health, stress, and lives. Most believed that this support would be beneficial in working out alternative strategies to deal with stress and loneliness. These could involve counselling and participants focusing on establishing greater social connections.

Participants recommended that part of this support should focus on finding alternative ways to deal with stress before quitting smoking and preparing people to deal with the impact of quitting, such as losing a way to connect with others and giving meaning to the day.

That smoking has a place, like it's not something to be ashamed of or embarrassed about. It's actually something similar to taking medication or drinking coffee. It has a biological impact on your life, and because of that, it can serve a function. So you're not attacking the person or making somebody feel ashamed or embarrassed because it's something that they do but helping them to appreciate that it has a function in their lives, but also knowing that there are other things that we can do that have a physiological impact on us as well. So, helping to understand how smoking might have an impact on lowering your heart rate or relaxing, but other things can do that too. So rather than targeting smoking, being able to see it as one of many things that you can do that have the same benefit.

(Cameron)

I think just honing in on smoking and making somebody feel like there's something wrong, rather than you know, there are multiple ways that we can cope and live in the world, and smoking is a valid way of doing that. But they also let you know and support to see if there are other ways that you might like to do that as well, so that it might not have the harmful side effects that smoking has.

(Taylor)

Participants described alternative ways of reducing stress that included exercise and participating in peer support groups.

I started looking after myself more and started walking and attending groups where we socialised without smoking.

(Cameron)

Similarly, holistic approaches and addressing issues such as weight gain were seen to be important.

I got fatter again. And this is creating all kinds of health, disability, and access problems in my life. […] it would have been nice if there was some support around that. But they leave us alone with these impacts.

(Ash)

Some participants spoke about how they experienced the smoking ban in the hospital as being forced to quit smoking. For these participants, the support they were provided to quit during those times was perceived as inadequate. They believed that it had not been the right time to quit, and as soon as they left the hospital, they started to smoke again.

It [in hospital] is a shit time to quit. Yeah, you don't want to quit when you're, like, off your tree. Like it was one of the last few comforts that I had, you know, much bigger concerns about, you know, hearing, you know, terrifying voices, suicidality, really extreme stuff. The potential of getting lung cancer one day is so far down the list that it is ridiculous.

(Ash)

When participants were asked about what support would have been helpful during a mental health admission to assist with quitting smoking, they noted that more holistic support was needed, which included counselling rather than just nicotine replacement strategies.

[I would have liked] a little bit of counselling or whatever. Just so, when I got out of the hospital, I didn't go straight back to it like I did.

(Finley)

Participants also believed that smoking bans, without more systemic and holistic approaches to supporting people in these environments, were ineffective as smoking cessation measures. This was particularly the case for smoke‐free mental health inpatient units, which were often conflated with enforced quitting. To be effective, participants would prefer a combination of strategies that included, but were not limited to, nicotine replacement therapy and additional psychological approaches to manage causes of stress and anxiety.

No mental health professionals offered any support around quitting when I was at the hospital. They conflated it, I think, like smoking bans with quitting. I want them to try to stop their practices from being so awful. It's not the same thing at all. Being smoke‐free and not allowing us to smoke is not at all the same thing as giving us support with quitting.

(Ainsley)

One person suggested a harm‐reduction approach to smoking cessation. This involved learning from the alcohol and other drug sectors.

I would say learn from the harm reduction movement in Alcohol and Other Drugs, like, smoking stuff, has gone really hardcore down this prohibition path, it seems to me, and when it intersects with mental health, where there are already such extreme human rights violations, people don't seem to think twice about the use of coercion and even direct force. And I would say to people that's not only a grotesque violation of people's rights, but it's also not helpful. It's not helpful. Even if you do get us to quit smoking, you might, in the process, do really serious harm to us. We've already been betrayed so many of us so many times, and survived so many kinds of violence. So, I would say to people, get as far away from these models of coercion and threat and force and prohibition as you can and look to harm reduction.

(Ash)

Although there were no concrete examples of harm reduction approaches, this appeared to counter stricter approaches and smoking bans.

### 5.2 Quitting Smoking Is Related to Personal Growth

While financial, physical health and some social factors contributed to the participants' motivation to quit, personal growth and mental health recovery were the most compelling reasons cited by participants.

I did become conscious at about that time of being a role model as a peer, which has an aspirational nature that if others wanted to change, I was there as an example of the fact that change was possible, that I'd found a way to take back control of what was happening in my life, and that I believed they could do that too.

(Drew)

Once I'd made that decision, everything didn't just go, “Oh, woosh, fantastic.” It was really rocky, and there was a lot of doubt and times when I second‐guessed whether or not I was doing what was best, but I'd at least had that moment of totally showing up, backing myself, and saying, “You can do this,” and I drew a lot from that. I actually used that decision‐making and determination [to quit smoking] to check back in with myself and say I can remember when I was here, and I wanna make that choice and look at what's happened since I made that choice.

(Jamie)

Many participants considered their decision to stop smoking to be part of a process where they wanted to become a ‘better person’ or more ‘aligned with their values’. These moments were often linked to internal resolve and a turning point in their lives and mental health. Overall, recommendations for how health professionals should respond to mental health consumers who smoke highlighted the importance of compassion and responding without shaming people. The contrast between support for quitting and managing withdrawal symptoms as part of smoke‐free inpatient stays is significant in consideration of how consumers may perceive these as punitive and a barrier to personal recovery.

## 6 Discussion

This study focused on the experiences of smoking and quitting for people with a mental health diagnosis. For all participants, there was a strong relationship between smoking and their mental health, with many describing how smoking was a way to alleviate high levels of stress and anxiety, resulting in quitting smoking being perceived as more challenging than for those without mental health diagnoses. Collectively, the experience of smoking was a complex mix of positive and negative experiences. Many participants highlighted that smoking provided opportunities for connection with others who smoke but also segregated them from others due to smoke‐free policies limiting where they can smoke. Notably, while financial, health, and social factors were described as contributing factors in quitting, the most compelling reasons related to personal growth, and many equated quitting smoking with becoming ‘aligned with their values’.

The findings from this study add to emerging literature that indicates that while addressing physical withdrawal symptoms of smoking is important for people experiencing mental health distress, addressing the role of smoking in a person's life is equally important [12, 24]. Quitting smoking can be more difficult for this population as not only can the symptoms of nicotine withdrawal mimic symptoms of anxiety or depression, but smoking is often perceived as a way of managing these symptoms and as such, participants believed that smoking cessation could exacerbate these symptoms [7]. However, in reality, evidence suggests that quitting smoking may be associated with small to moderate improvements in mental health [25, 26]. Supporting people to find alternative ways to deal with stress, anxiety, and depression should be a priority during smoking cessation attempts or periods of enforced abstinence [27].

There will be benefits in campaigns targeted to this population that debunk the common perception that smoking generally helps people manage stress, with greater evidence suggesting that quitting may reduce stress and result in improvements in mental health symptoms in the longer term [4, 25]. It has been noted that people living with schizophrenia were significantly more likely to smoke to reduce stress compared with people from the general population and that pharmacological interventions for people living with a mental illness were only effective for less than 20% of people who smoke [27]. Participants expressed an interest in receiving counselling, which is supported by literature that provides both Nicotine Replacement Therapy and counselling for smoking cessation, which is important to deal with the social and psychological aspects of quitting smoking in addition to physical withdrawal [28].

The timing of smoking cessation support is also critical. Embedding cessation support as routine practice in all mental health and healthcare settings and offering smoking cessation support at different points in a person's recovery journey could be beneficial. The desire to quit and the sense of accomplishment people experience when they stop smoking highlight the importance of repeatedly offering smoking cessation support in a non‐judgmental way [29]. Timing may be related to ambivalence in an individual's beliefs, affecting readiness in deciding to quit [30]. Ambivalence related to quitting smoking can be very common, and motivation often fluctuates. Our findings corroborate those of others, which suggest that this experience may be particularly strong for people diagnosed with a mental illness who often experience self‐doubt and self‐stigma [12].

Desired smoking cessation from participants supports the use of compassionate smoking cessation support, delivered in a non‐judgmental manner with a focus on the role smoking has in a person's life, which may increase motivation to quit among people experiencing mental illness [12]. The literature suggests that quit smoking support would benefit from instilling hope, confidence, and self‐belief to try quitting in the future and highlighting how quitting smoking may assist individuals in their internal resolve to better themselves. In‐depth conversations around the reasons why people smoke were generally experienced as clarifying and motivating and could be the start of someone's decision to quit smoking [12].

It is important to address low rates of Nicotine Replacement Therapy and low expectations of quitting held by mental health staff [29]. Smoking cessation that facilitates the hope and self‐belief that people with a mental health diagnosis can quit is significant when people, because of overwhelming mental health symptoms, may have returned to smoking to be able to manage these [12]. Specialised smoking cessation treatment may be needed that is tailored to people with a diagnosis of mental illness [31]. This support includes not only having smoking cessation expertise but also openness to understanding the perceived value of smoking in helping to manage psychological distress and supporting strategies that promote personal recovery.

## 7 Conclusion

People experiencing mental ill health face unique challenges when attempting to quit smoking. This study suggests that people diagnosed with a mental illness, with the right support and timing, are most likely to be willing and able to quit smoking. While overarching strategies such as smoking cessation should involve behavioural as well as pharmacological support to address the impact of cravings, this study highlights that these need to be delivered in a recovery‐focused way, which enhances self‐determination and non‐judgmental support to make the decision to quit smoking.

## Author Contributions

Helena Roennfeldt led the writing, analysis and revision of the final paper; Marianne Wyder contributed to the development of the design of the study, analysis and writing of the initial draft and revisions; Coral Gartner contributed to the design of the study and revisions; Alice Holland contributed to the revisions, Norah Elvidge assisted with data collection and Cheneal Puljevic led the study design and contributed to revisions.

## Disclosure

The authors have nothing to report.

## Ethics Statement

The study was approved by Metro South Health's Human Research Ethics Committee (HREC/2020/QMS/70453).

## Conflicts of Interest

The authors declare no conflicts of interest.

## Data Availability

The data that support the findings of this study are available on request from the corresponding author. The data are not publicly available due to privacy or ethical restrictions.
